# Injection Molding of Magnesium Aluminate Spinel Nanocomposites for High‐Throughput Manufacturing of Transparent Ceramics

**DOI:** 10.1002/advs.202204385

**Published:** 2022-09-04

**Authors:** Markus Mader, Richard Prediger, Karl G. Schell, Gabriela Schmidt, Alex Dorn, Sophie Jenne, Sebastian Kluck, Leonhard Hambitzer, Manuel Luitz, Claudia Schwarz, Marcel Milich, Christian Greiner, Bastian E. Rapp, Frederik Kotz‐Helmer

**Affiliations:** ^1^ Laboratory of Process Engineering NeptunLab Department of Microsystems Engineering (IMTEK) Albert Ludwig University of Freiburg 79110 Freiburg Germany; ^2^ Freiburg Materials Research Center (FMF) Albert Ludwig University of Freiburg 79104 Freiburg Germany; ^3^ Institute for Applied Materials (IAM) Karlsruhe Institute of Technology (KIT) 76131 Karlsruhe Germany; ^4^ IAM‐KWT Ceramic Materials and Technologies Haid‐und‐Neu Strasse 7 76131 Karlsruhe Germany; ^5^ Institute of Physical Chemistry Albert Ludwig University of Freiburg 79104 Freiburg Germany; ^6^ Institute for Macromolecular Chemistry Albert Ludwig University of Freiburg 79104 Freiburg Germany; ^7^ Gisela and Erwin Sick Chair of Micro‐optics Department of Microsystems Engineering (IMTEK) Albert Ludwig University of Freiburg 79110 Freiburg Germany; ^8^ Hahn Schickard Georges‐Köhler‐Allee 103 79110 Freiburg Germany; ^9^ Electrochemical Energy Systems Department of Microsystems Engineering (IMTEK) Albert Ludwig University of Freiburg 79110 Freiburg Germany; ^10^ IAM‐ZM – MicroTribology Center µTC Kaiserstrasse 5 76131 Karlsruhe Germany; ^11^ Glassomer GmbH Georges‐Köhler‐Allee 103 79110 Freiburg Germany; ^12^ FIT Freiburg Center of Interactive Materials and Bioinspired Technologies Albert Ludwig University of Freiburg 79110 Freiburg Germany

**Keywords:** injection molding, magnesium aluminate spinel, thermoplastic nanocomposite, transparent ceramic

## Abstract

Transparent ceramics like magnesium aluminate spinel (MAS) are considered the next step in material evolution showing unmatched mechanical, chemical and physical resistance combined with high optical transparency. Unfortunately, transparent ceramics are notoriously difficult to shape, especially on the microscale. Therefore, a thermoplastic MAS nanocomposite is developed that can be shaped by polymer injection molding at high speed and precision. The nanocomposite is converted to dense MAS by debinding, pre‐sintering, and hot isostatic pressing yielding transparent ceramics with high optical transmission up to 84 % and high mechanical strength. A transparent macroscopic MAS components with wall thicknesses up to 4 mm as well as microstructured components with single micrometer resolution are shown. This work makes transparent MAS ceramics accessible to modern high‐throughput polymer processing techniques for fast and cost‐efficient manufacturing of macroscopic and microstructured components enabling a plethora of potential applications from optics and photonics, medicine to scratch and break‐resistant transparent windows for consumer electronics.

## Graphical abstract

The development of thermoplastic magnesium aluminate spinel (MAS) nanocomposites allows the fabrication of 3D, transparent MAS with single micron resolution using high‐throughput polymer injection molding. The injection molded nanocomposites are subsequently converted to dense and transparent MAS using debinding, pre‐sintering, and hot isostatic pressing. The injection molded MAS shows high optical transparency, low surface roughness, and high mechanical stability.

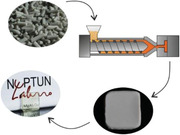

## 1 Introduction

Transparent polycrystalline ceramics like magnesium aluminate (MgAl_2_O_4_) spinel (MAS) ceramics are of great academic and industrial interest due to their unique material properties.^[^ ^1^, ^2^, ^3^ ^]^ Excellent mechanical and thermal stability combined with a low thermal expansion coefficient, high chemical and radiation resistance as well as good optical properties with transmission > 85% over a wide range from 250 nm in the ultraviolet to 6500 nm in the infrared, make MAS ceramics the material of choice for various applications ranging from infrared domes and highly resistant cover windows ^[^ ^4^, ^5^, ^6^ ^]^ to solid‐state lasers,^[^ ^7^ ^]^ medical applications such as orthodontic brackets ^[^ ^8^ ^]^ and insulating materials in extremely harsh environments such as in fusion reactors.^[^ ^9^ ^]^ Transparent MAS is especially interesting for optical applications since the ceramic features not only high transparency but also combines a high refractive index (*n*_532 nm_ = 1.7108) with a low optical dispersion (Abbe number ≈60) enabling the fabrication of thin, optical components displaying low chromatic aberration for, e.g., ultra‐thin high‐quality camera lenses.^[^ ^10^, ^11^ ^]^ Although this combination is inaccessible in other transparent commodity materials such as polymers or glasses, high refractive indices are always coupled to high dispersions due to their amorphous nature in these materials.^[^ ^12^ ^]^

Despite its beneficial properties, MAS is not used as commonly as for example transparent glasses or polymers. This is due to the fact that the extreme hardness and the high chemical and thermal stability make structuring of transparent MAS ceramics inherently challenging. Transparent MAS ceramics are nowadays mainly fabricated using casting of MAS powders from water‐based solutions or by pressing of the raw powders followed by a sintering process.^[^ ^1^, ^3^, ^13^ ^]^ The sintering of polycrystalline MAS to full density, transparent ceramics has been studied and discussed at length with the best results being usually achieved using either spark plasma sintering (SPS) or hot isostatic pressing (HIP).^[^ ^1^, ^2^, ^3^, ^13^, ^14^, ^15^ ^]^ This, however, can only achieve simple geometries such as flat plates or domes requiring complex and time‐consuming post‐processing steps such as grinding, polishing, or etching for more complex features.^[^ ^1^, ^3^, ^13^ ^]^

Recently, additive manufacturing of transparent MAS ceramics has been introduced allowing fabrication of transparent 3D MAS ceramics by stereolithography printing of a liquid nanocomposite followed by sintering and HIP.^[^ ^16^ ^]^ Further, direct 3D printing of transparent MAS has been shown using a laser deposition method inspired by the *Verneuil* method, which, however, suffers from prevalent cracking.^[^ ^17^ ^]^ However, due to the layer‐based nature of 3D printing the printed components are inherently rough and excessive post‐processing is necessary for the fabrication of optical components. While these methods enable 3D structuring of transparent MAS, these processes are not suited for mass‐market manufacturing, which can only be achieved by high‐throughput manufacturing methods such as injection molding. Powder injection molding (PIM) is a well‐known method for manufacturing inorganic components based on thermoplastic composite materials that are shaped using polymer injection molding and then converted to dense components by subsequent heat treatments.^[^ ^18^, ^19^, ^20^ ^]^ PIM has already been shown for several inorganic materials such as fused silica glass,^[^ ^21^ ^]^ metals such as steel ^[^ ^22^ ^]^ and titanium ^[^ ^23^ ^]^ or ceramics such as zirconia ^[^ ^24^ ^]^ and alumina.^[^ ^25^ ^]^ However, one of the most interesting transparent ceramics, MAS, has so far not been accessible to high‐throughput injection molding.

In this work, we present thermoplastic MAS nanocomposites that can be shaped and structured using conventional injection molding machinery. The injection molded components are debinded using a two‐step process followed by sintering at 1550 °C to almost full density (> 97%). Fully densified and transparent ceramics were obtained after HIP treatment at 1700 °C under argon pressure (1500 bar). The HIP‐treated MAS components show high optical transmission of up to 84 % as well as high surface quality (roughness *R*_q_ < 20 nm) without any further post‐processing steps. Besides complex shaped macroscopic parts this process allows microstructuring of transparent MAS ceramics with high resolution allowing replication of structure with single micrometer‐sized features. This process enables manufacturing of complex shaped, transparent MAS ceramics by injection molding rendering an outstanding high‐performance material available to the mass market with possible applications ranging from transparent high‐stability components for harsh environments to small‐scale, high‐performance optics.

## 2 Preparation and Processing of Thermoplastic MgAl2O4 Nanocomposite

**Figure 1:**
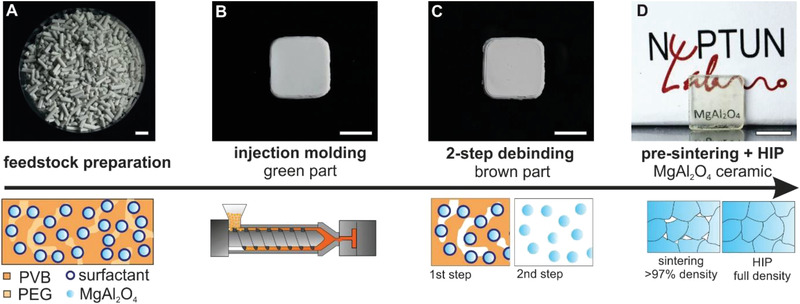
Injection molding of transparent MAS ceramics. A) Pre‐mixing MAS nanopowders with thermoplastic polymers allows the preparation of thermoplastic MAS nanocomposite granules. Scale bar: 10 mm. B) The granules can be processed by injection molding at mild temperatures ≈140 °C resulting in the green part. Scale bar: 10 mm. C) The green part is debinded using a two‐step debinding procedure, consisting of liquid debinding of PEG and subsequent thermal debinding of residual binder components yielding defect free brown parts. Scale bar: 10 mm. D) The brown parts are pre‐sintered at 1550 °C to a density > 97% with closed porosity followed by HIP treatment to obtain transparent MAS components with full density. Scale bar: 10 mm.

We prepared a thermoplastic MAS nanocomposite by pre‐mixing MAS nanopowders having a specific surface area of 26.7 m^2^ g^–1^ (BET) with thermoplastic binder components. Transmission electron microscopy (TEM) was used to analyze the size and morphology of the MAS nanopowders (Figure S1A, Supporting Information). The TEM images show roughly spherical shaped particles with a diameter of ≈50 nm. The composition of the thermoplastic binder systems was tailored to optimal thermoplastic processing behavior considering miscibility of the binder components as well as wettability of the MAS nanopowders for homogenous, highly filled nanocomposites. Polyvinyl butyral (PVB) was chosen as backbone binder component since its high polarity suggests a good compatibility with inorganic and polar particle surfaces. Polyethylene glycol (PEG) was added, first as plasticizer to reduce melt viscosity, and second as an intermediary phase enabling a preliminary solvent‐based debinding step that is essential for thicker components. To improve powder wettability a surfactant was added as third binder component. Polyacrylic acid, combining high acid group density, high polarity, and good mechanical stability showed the best results and was therefore chosen as a surfactant. To prepare the MAS nanocomposite, the respective binder components were premixed with the MAS nanopowders in solution to achieve high homogeneity. After evaporating the solvent, the obtained pre‐mixed feedstock was plasticized at 140 °C, extruded and granulated to obtain nanocomposite granules (**Figure** **1**A). Optimization of the binder composition allowed to reach MAS solid loadings of up to 40 vol.% that could still be processed using standard polymer extrusion and injection molding machinery. High solid loadings are typically favored, since it reduces shrinkage and increases sinterability and densification. However, high solid loadings also increase melt viscosity making processing more difficult. Therefore, a balance has to be found between highest possible solid loading while still retaining low viscosity for successful processing. Increasing the solid loading beyond 40 vol.%, which would benefit sinterability even further, led to a significant increase in material viscosity and total loss of the materials processability. At a temperature of 140 °C, the feedstock containing 40 vol.% MAS nanopowders exhibits a viscosity < 1500 Pa s at a typical injection molding shear rates of ≈10^3^ s^–1^, as determined using a capillary rheometer (Figure S1B, Supporting Information). The feedstock with a lower MAS solid loading of 35 vol.% showed a viscosity of ≈1000 Pa s (Figure S1B, Supporting Information). Both MAS feedstocks show strong shear thinning behavior but the viscosities at high shear rates still lie in the upper region of commonly recommended viscosities for successful powder injection molding (< 1000 Pa s).^[^ ^26^, ^27^ ^]^ Despite the rather high viscosities the nanocomposites could be processed using injection molding without facing difficulties such as incomplete mold filling or shear‐induced molding defects. The thermoplastic MAS feedstock was injection molded at mild temperatures of 140 °C and specific injection pressure of up to 700 bar resulting in mechanically stable green parts (Figure 1B). These green parts were then immersed in water to dissolve the PEG component in a first debinding step. The duration of the aqueous debinding step depends on the thickness of the processed component. For components where the green part had at least 1D that was <2 mm the PEG component could be fully removed within 4 h in water with a temperature of 40 °C (Figure S1C, Supporting Information). For components with a wall thickness of up to ≈6 mm the aqueous debinding duration was increased to 8 h. Residual binder components were removed by thermal decomposition in a second debinding step yielding defect‐free brown parts (Figure 1C). We optimized the process parameters of the thermal debinding by evaluating thermal gravimetric analysis (TGA) measurements of aqueous debinded components (Figure S1D, Supporting Information). Dwelling phases of 1 h each were set at critical temperatures of the polymer decomposition (270, 400, and 600 °C). Due to the porosity generated by the aqueous debinding step heating rates of 1 K min^–1^ could be employed without observing any cracks after debinding. The debinded samples were then pre‐sintered at 1550 °C to a density of at least 97% to ensure closed porosity. The increase in solid loading has shown a substantial advantage in sintering the MAS ceramics to high densities. The 40 vol.% nanocomposite reaches a density of >> 97% without any dwelling time at 1550 °C, while the 35 vol.% nanocomposites already need a minimum of 90 min dwelling time at 1550 °C to reach ≈97 % density. This effect increases with further decreasing solid loadings. A 30 vol.% nanocomposite cannot be sintered to > 97 % density within a reasonable dwelling time of 4 h.

Grain growth during sintering was characterized using scanning electron microscopy (SEM) on thermally etched samples (Figure S2, Supporting Information). We found small mean grain diameters of 0.9 ± 0.3 µm for a 35 vol.% feedstock and 1.3 ± 0.5 µm for a 40 vol.% feedstock. Both compositions showed a narrow size distribution without abnormal grain growth occurring, which is mandatory to allow full densification in a post‐sintering process such as HIP. It has been shown, that abnormal grain growth usually leads to very stable microstructures limiting the mobility of grains during HIP and therefore preventing the elimination of residual porosity.^[^ ^28^, ^29^ ^]^ In addition, the SEM images reveal several remaining pores in the pre‐sintered not yet fully densified ceramic (Figure S2C, Supporting Information). The pre‐sintered MAS ceramics were then HIP treated in an argon atmosphere for 2 h at a temperature of 1700 °C and 1500 bar pressure. After the HIP treatment, the MAS ceramics reached full density (3.584 ± 0.005 g cm^–3^, 100.1 ± 0.2 %, Table S1, Supporting Information). SEM images show that the residual pores found in the pre‐sintered MAS (Figure S2C, Supporting Information) have been eliminated (Figure S2D, Supporting Information). After HIP treatment the fully densified MAS parts became transparent, although showing a slight discoloration (Figure S3A,B, Supporting Information). This discoloration is well‐known in HIP treatment of transparent ceramics and it was proposed that this effect results from oxygen deficiency. The graphite heaters of the HIP system generate a slightly reducing atmosphere during the HIP treatment causing a minor shift in the MAS ceramics stoichiometry.^[^ ^30^, ^31^, ^32^ ^]^ To restore the stoichiometry and remove the discoloration the HIP treated samples were heated in air atmosphere at a temperature of 1200 °C for 2 h yielding colorless transparent ceramics (Figure 1D). Scanning electron microscopy – energy dispersive X‐ray spectroscopy (SEM‐EDX) measurements were conducted to analyze the elemental composition and possible carbon contamination of the HIP treated MAS components before and after the additional heat treatment to remove the discoloration (Figure S3C, Supporting Information). No substantial differences could be observed between the two spectra showing strong signals for MAS and no significant carbon signals in either measurement. A quantitative analysis of the elemental composition (Table S2, Supporting Information) yielded an elemental composition of ≈14% magnesium, 29% aluminum, and 52% oxygen being close to the expected MAS composition (MgAl_2_O_≈3.7_). A quantitative analysis of possible carbon contamination yielded large errors indicating that the real content is below the detection limit of the system. During the HIP treatment grain growth is suppressed and while it is possible to achieve full density only a small increase in the grain size and grain size distribution was observed (Figure S4, Supporting Information), with no substantial influence on the initial nanocomposites solid loading. The HIP treated transparent MAS components showed grain sizes with a mean diameter of ≈5 ± 2 µm for a 35 vol.% feedstock and 6 ± 3 µm for a 40 vol.% feedstock.

**Figure 2:**
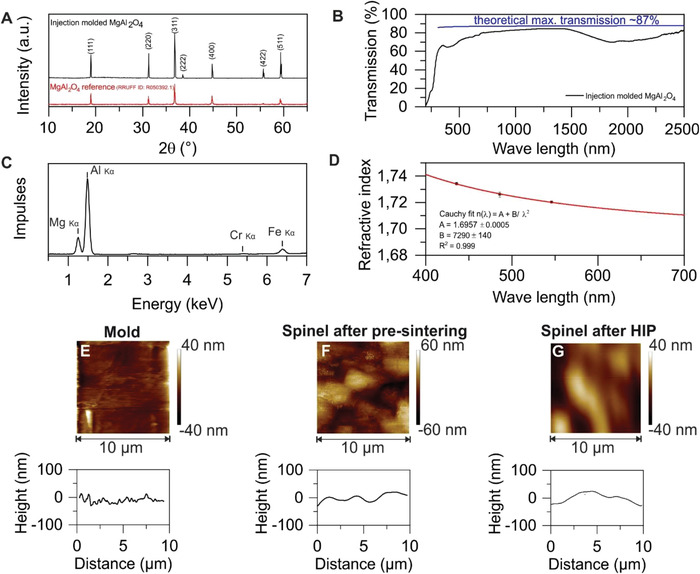
Characterization of injection molded and HIP treated MAS. A) XRD measurement of a transparent MAS component compared to a reference diffractogram (RRUFF ID: R050382.1). B) After the HIP treatment the injection molded MAS ceramic (thickness 1.8 mm) shows high transmission of up to 84% in the UV–vis and NIR region. C) XRF measurement of injection molded transparent MAS showing high purity MAS ceramics with only minor Cr and Fe contaminations. D) Wavelength‐dependent refractive indices of the transparent MAS ceramic. The measured indices (average with standard deviation, min. 9 measurements) are shown as black squares and the respective *Cauchy* fit is shown as red line. AFM measurement of E) a mold, F) a pre‐sintered MAS and G) transparent MAS after HIP treatment showing that high surface qualities (roughness *R*_q_ < 20 nm) can be achieved if molding tools with optical surface finish are used.

X‐ray diffraction (XRD) measurement was performed and compared to a reference diffractogram confirming that the crystal structure of the transparent ceramic obtained after HIP treatment was pure MAS (**Figure** **2**A). During the sintering and HIP steps the MAS components show an isotropic shrinkage from nanocomposite green part to final transparent ceramic. This shrinkage can be calculated theoretically in dependence of the solid loading according to Equation (2). For an exemplary sample injection molded using a 35 vol.% feedstock, we measured a linear shrinkage of 29.52 ± 0.31 % in *z* and 29.53 ± 0.08 % in *x* and *y* directions (Table S3, Supporting Information), which is in good accordance to the calculated value of 29.53%.

The obtained transparent MAS ceramic components show exceptionally high thermal and chemical resistance. The MAS ceramics are able to withstand high temperatures up to 1200 °C over a long duration of at least 48 h without changes to the component dimensions or optical transparency. At temperatures of > 1300 °C up to at least 1550 °C the MAS ceramics still retain dimensional stability but quickly (≈1 h) lose their optical transparency, due to grain growth and expansion of pores, making the ceramics become opaque.^[^ ^31^, ^32^ ^]^ The MAS ceramics high chemical resistance was shown by immersing the components in various common acidic, basic, or organic chemicals for 24 h showing no visual or measurable effect in weight on the ceramic components (see Table S4, Supporting Information).

### 2.1 Mechanical and Optical Analysis of Transparent MAS Prepared by Injection Molding and HIP Treatment

Micro‐Vickers Hardness *HV0.1* and bending strength were measured to characterize the mechanical properties of the resulting MAS samples. The injection molded and HIP treated MAS show a high Micro‐Vickers Hardness of 1860 ± 120 HV or 18.2 ± 1.2 GPa (Table S6, Supporting Information) that lies within the upper region of reported literature data.^[^ ^1^, ^13^, ^33^ ^]^ The high hardness can be attributed to the low grain sizes and the absence of sintering aids since it has been shown repeatedly that dopants, as well as large grain sizes, lead to decreasing hardness of MAS ceramics.^[^ ^33^, ^34^ ^]^ We additionally measured a commercial single‐crystal MAS substrate as reference (1500 ± 20 HV, 14.7 ± 0.2 GPa, Table S6, Supporting Information) demonstrating that the fine‐grained microstructure of injection molded and HIP treated MAS leads to a significant increase of the hardness compared to the single‐crystal substrate. In addition, the bending strength of transparent injection molded and HIP treated MAS was measured via three‐point bending. The transparent MAS ceramic shows a high bending strength of 164 ± 40 MPa (Table S7, Supporting Information), which is in good accordance to previously reported data for MAS ceramics prepared by various alternative preparation methods (≈110–180 MPa).^[^ ^35^, ^36^ ^]^

The sintered and HIP treated MAS parts show a high transparency from 300 to 2500 nm as determined by UV–vis‐NIR total transmission measurement using a 1.8 mm thick sample (Figure 2B). The theoretically calculated maximum transmission (calculation according to Equation (3) and (4)) is shown as reference. The transparent MAS ceramic shows a high transmission close to the theoretical maximum with up to 84 % transmission at 1000 nm (theoretical maximum at 1000 nm = 87.3 %) similar to previous reports on transparent MAS ceramics.^[^ ^16^, ^17^, ^28^, ^33^, ^37^, ^38^ ^]^ A slight absorption can be observed in the upper NIR region reducing the transmission by 10% at most. This absorption may be caused by Fe^2+^ impurities, probably due to non‐hardened steels employed in the injection molding process. It has been shown that iron doping concentrations as small as 0.1 mol.% yields strong NIR absorption peaks substantially larger than the ones observed in the spectrum shown in Figure 2B suggesting a significantly lower impurity concentration within our injection molded MAS.^[^ ^39^, ^40^ ^]^ For a quantitative analysis of the impurity concentration we characterized the HIP treated MAS using X‐ray fluorescence (XRF) as shown in Figure 2C. We found weak signals for chromium and iron impurities, most likely due to minor abrasion during compounding and injection molding. We found that the amount of chromium and iron impurities is <70 ppm (Cr) and 430 ppm (Fe), respectively (Table S5, Supporting Information). Fourier transform infrared spectroscopy (FTIR) measurements were conducted to study the maximum window of transparency reaching into the mid‐infrared region from 3500 to 7000 nm (Figure S3D, Supporting Information). The transparent MAS shows high transmissions of ≈80% up to 5000 nm, transmissions >60% at 5500 nm and finally reaching 0% transmission at 6700 nm, comparable to previously reported data on transparent MAS.^[^ ^1^, ^14^, ^41^ ^]^ We performed refractive index measurements and determined the optical dispersion by calculating the Abbe number *ν*. Refractive index values were measured at 435.8, 486.1, and 546.1 nm. We found high refractive indices in the range of 1.7341 to 1.7201 (Table S8, Supporting Information) that is in good accordance to typically reported refractive indices for transparent MAS (n≈1.73‐1.72).^[^ ^1^, ^14^ ^]^ To calculate the Abbe number according to Equation (6) we approximated the refractive index over the visible region (400–700 nm) by fitting the measurements using a simplified version of the *Cauchy* equation (Equation 5), which has been shown to be a good approximation of the refractive index in the visible region.^[^ ^24^, ^42^ ^]^ The measured indices and the *Cauchy* fit of HIP treated MAS are shown in Figure 2D. The measured, fitted, and theoretically calculated refractive index values are given in Table S8 (Supporting Information). The fitted refractive indices agree well with the theoretical values yielding, e.g., *n*_d,20_ = 1.7168 ± 0.0006 for the injection molded and HIP treated MAS matching the theoretically calculated value of 1.7162. A high Abbe number of *ν* = 52 ± 4 was determined from the fitted data that is in good accordance to commonly reported values for transparent MAS (*ν*≈60^[^ ^1^ ^]^) showing that the injection molded and HIP treated MAS combines high refractive index and low optical dispersion.

The surface quality of the transparent MAS ceramics prepared by injection molding was analyzed using atomic force microscopy (AFM). Since there is no smoothening of surfaces during the sintering process, the surface quality of the injection molded and sintered component is mostly determined by the surface quality of the employed molding tool.^[^ ^21^ ^]^ Using an injection mold with a surface roughness of *R*_q_ < 20 nm (measured on an area of 100 µm^2^, Figure 2E) we were, therefore, able to manufacture transparent MAS ceramics with smooth surfaces without the need for polishing. AFM measurements of the transparent MAS parts show a surface roughness of *R*_q_ = 20 nm after pre‐sintering (measured on an area of 100 µm^2^, Figure 2F) and *R*_q_ = 14 nm after additional HIP treatment (measured on an area of 100 µm^2^, Figure 2G). Additional surface characterization of injection molded and HIP treated MAS was performed using white light interferometry on a larger area yielded a surface roughness of *S*_q_ = 148 nm (measured on an area of 0.12 mm^2^, Figure S3E, Supporting Information).

### 2.2 Manufacturing of Macroscopic and Microstructured Transparent MAS

**Figure 3:**
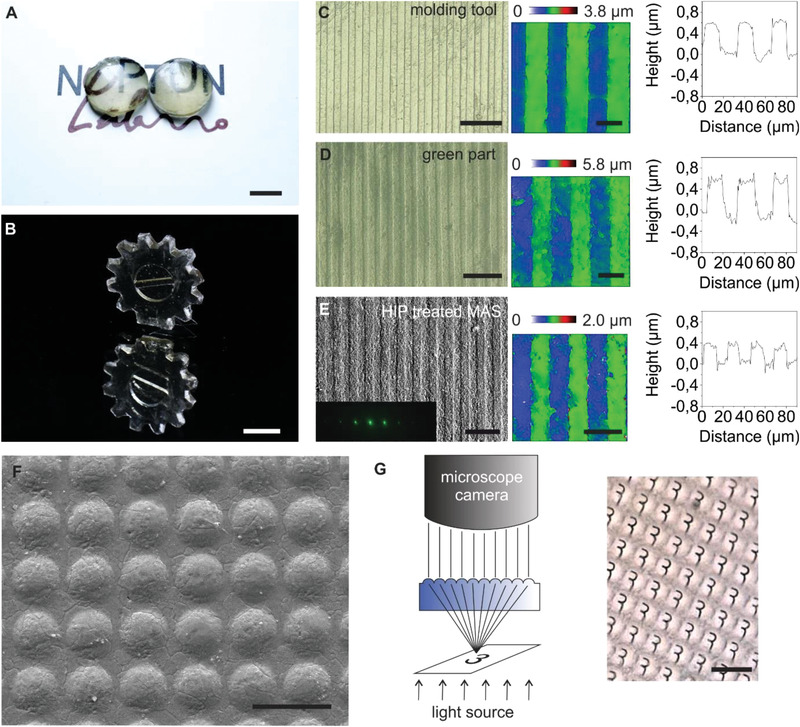
Macro‐ and microstructuring of transparent MAS ceramics by injection molding. A,B) Exemplary injection molded macroscopic lenses and a gear that were sintered and HIP treated to obtain full density, transparent MAS. Scale bar: 5 mm. C) Microscopy image (scale bar: 100 µm) of a microstructured mold inset showing a diffractive linear grid structure as well as white light microscopy image (scale bar: 20 µm) and the respective height profile. D) Microscopy image (scale bar: 100 µm) of MAS nanocomposite green part replicated from the mold shown in (C) using injection molding as well as white light microscopy image (scale bar: 20 µm) and the respective height profile. E) Scanning electron microscopy (SEM) image (scale bar: 50 µm) of transparent MAS prepared by pre‐sintering and HIP treatment of the green part shown in (D) as well as white light microscopy image (scale bar: 20 µm) and the respective height profile. The inset shows the characteristic diffraction pattern of the line grid by illuminating the transparent microstructured MAS with a green laser (532 nm). F) SEM image of an injection molded spherical MLA in transparent MAS with lens diameters of ≈32 µm. Scale bar: 50 µm. G) The optical functionality of the MLA was demonstrated by focusing through the lenses on an exemplary number “3” placed underneath the microstructured component. Scale bar: 50 µm.

We fabricated exemplary transparent, more complex shaped MAS components such as a lens and a gear demonstrating that functional, macroscopic components with wall thicknesses up to at least 4 mm can be produced by injection molding of the MAS nanocomposite and converted to transparent MAS ceramic by subsequent heat treatment (**Figure** **3**A,B). The spherical MAS lens has a curvature radius of 11.1 mm (calculation according to Equation (8), with *r* =5.75 mm and *h* = 1.6 mm) resulting in a focal length of *f* = 15 mm (calculation according to Equation (7)). It has to be noted that thicker components such as the lens (> 4 mm wall thickness) still retained a slight yellow discoloration even after the additional thermal treatment to restore oxygen stoichiometry. This is caused by the fact that the oxygen restoration is a diffusion‐based process that slows down with increasing sample thickness.

We evaluated several molding tool materials without noticing an influence on the final quality of the transparent ceramic. Molds made out of steel, brass, and aluminum have been used for injection molding of the nanocomposite (Figure S5A–C, Supporting Information). Small series or prototypes can be quickly and easily injection molded using polymer molding tool inserts made by stereolithography printing of commercial tooling resins (e.g., Asiga Fusion Gray) or by soft replication of master structures with commercial epoxy resins (Figure S5D,E, Supporting Information).

Besides macroscopic structures, this PIM process also allows facile microstructuring of transparent MAS ceramics with micrometer resolution enabling potential high‐throughput fabrication of, e.g., micro‐optical structures that are of high interest for the next generation of thin high‐performance optics. Micro‐optical features with single micrometer‐sized structures were replicated precisely in transparent MAS ceramic despite their feature size being in a similar range as the grain size of the sintered and HIP treated ceramic. Figure 3C–E displays exemplarily the fabrication of a microstructured transparent MAS ceramic showing a diffractive linear grid from mold insert, green part to sintered and HIP treated transparent MAS. No changes to the microstructure despite the expected isotropic sintering shrinkage could be observed. White light microscopy was used to determine the feature sizes of the replicated line grid microstructure showing high‐resolution microstructures with a line width of ≈11 µm and a line height of ≈0.4 µm after HIP treatment. The smallest line grid microstructure successfully replicated this way in transparent MAS showed line widths of ≈6 µm after HIP treatment (Figure S3F, Supporting Information). We further fabricated an injection molded transparent MAS microlens array (MLA) with lens diameters *d* of ≈32 µm (Figure 3F). The lens center height *h* was determined by white light interferometry measurements (Figure S6A,B, Supporting Information, *h*≈4 µm). Calculations according to Equation (7) and (8) yield a lens curvature radius *R* of ≈130 µm and a respective focal length *f* of ≈180 µm. The surface roughness of the microlenses was measured to be *R*_q_ ≈150 nm (Figure S6C, Supporting Information), which is in good accordance to the previously determined surface roughness of the microstructured molding tool used in this process (*R*_a_ ≈140 nm).^[^ ^21^ ^]^ The optical functionality of the MLA was demonstrated by imaging an exemplary structure (the number “3”) by focusing through the lenses on the feature placed underneath the MLA as schematically shown in Figure 3G.

## 3 Conclusion

The development of novel thermoplastic MAS nanocomposites allows fabrication of complex shaped macroscopic as well as microstructured MAS components using powder injection molding for high‐throughput manufacturing. The nanocomposites are converted to MAS components with full density and high transparency by subsequent debinding, pre‐sintering, and HIP treatment. High transmissions of up to 84 % and high surface quality with a surface roughness of *R*_q_ < 20 nm, as needed for optical applications, can be achieved without further post‐processing steps. This enables fast and efficient shaping of complex, 3D, transparent MAS components using industrially scalable injection molding. This work paves the way to a large number of novel applications from optics and photonics to near indestructible products such as functional cover windows for consumer electronics and highly resistant medical products such as orthodontic brackets, that were previously inaccessible or too expensive on a larger scale due to the lack of suitable, high‐throughput MAS processing methods.

## 4 Experimental Section

### Materials

Polyvinyl butyral (PVB) was purchased from Kuraray Europe GmbH. Polyethylene glycole (PEG), polyacrylic acid, and ethanol (99.6 %) were purchased from Sigma–Aldrich. MAS nanopowders (S30CR) were purchased from Baikowski.

### MAS Nanopowder Characterization

The specific surface area of the nanopowder was measured by nitrogen adsorption according to the Brunauer–Emmet–Teller (BET) method using a Sorptomatic 1990 (Thermo Electron Corporation, USA). The specific surface area was automatically calculated using the three parameters fit method. The particle size and morphology were characterized using transmission electron microscopy (TEM) of type Talos 200x (Thermo Fisher Scientific, USA). The TEM sample was prepared by applying a small drop of an aqueous dispersion of the nanoparticles on a carbon film coated copper grid.

### Feedstock Preparation

Up to 40vol.% MAS nanopowders were premixed with the thermoplastic binder components. At first, the surfactant, polyacrylic acid (PAA, 15vol.% of organic binder), was dissolved in ethanol and the MAS nanopowders were added to the solution. The mixture was stirred using a laboratory dissolver of type RZR 2101 (Heidolph Instruments, Germany) for 30 min. Then, PVB (25.5vol.% of organic binder) and PEG (59.5vol.% of organic binder) were added to the suspension followed by stirring for another 30 min. The mixture was dried to obtain the pre‐mixed feedstock as a powder. The powder was then plasticized and extruded using a twin‐screw compounder of type DSM X‐plore 5.5 cm^3^ (X‐plore, Netherlands) at a temperature of 140 °C followed by granulation of the extruded nanocomposite strand using a granulator of type M50/80 (Hellweg, Germany).

### Injection Molding

The nanocomposite granules were plasticized in a compounder of type DSM X‐plore 5.5 cm^3^ (X‐Plore, Netherlands) at 140 °C. Subsequently, the plasticized material was injection molded using a microinjection molder of type DSM X‐Plore 4.5 cm^3^ (X‐Plore, Netherlands) at 140 °C with up to 700 bar injection pressure for 2 s and a holding pressure of 700 bar pressure for 3 s. Mold temperature was set to 50 °C for metal molds and room temperature for polymer molds.

### Debinding

The injection molded green parts were debinded in a two‐step process. In the first step, the parts were immersed in DI water at 40 °C for a minimum of 4 h to remove the water‐soluble PEG component. After drying the aqueous debinded parts at 75 °C under atmospheric pressure for a minimum of 3 h the residual binder was removed by thermal decomposition in air using an ashing furnace of type AAF (Carbolite/Gero, Germany). For the thermal debinding step heating rates of 1 K min^‐1^ were applied and dwelling phases of 1 h were set at critical decomposition temperatures (270, 400, and 600°C, as determined using TGA measurements).

### Pre‐Sintering and Hot Isostatic Pressing

The debinded samples were sintered to a density >97% using a high‐temperature bottom loader furnace of type BLF 18/3 (Carbolite/Gero, Germany). The 35vol.% feedstock was sintered for 3 h at 1550 °C and the 40vol.% feedstock was sintered for 15 min at 1530 °C with heating and cooling rates of 5 K min^‐1^, respectively. The pre‐sintered samples were fully densified using a hot isostatic press of type QIH‐6 (Asea, Ohio, USA) for 2 h at 1700 °C under argon pressure of 1500 bar with heating and cooling rates of 10 K min^–1^. Slight discolorations due to the hot isostatic pressing (HIP) process were removed by heating the HIP treated MAS in air to a temperature of 1200 °C for 2 h.

### Characterization of the Feedstock

The apparent viscosity of thermoplastic MAS feedstocks containing 35vol.% and 40vol.% solid loading was determined using a capillary rheometer of type Rheograph 25 (Göttfert, Germany). The capillary die used for the measurement had a diameter of 1 mm and a length of 10 mm. The measurement was performed at a temperature of 140 °C. Before the measurement, the material was allowed to rest in the barrel for a period of at least 10 min allowing the material to completely melt and reach thermal equilibrium. Shear rates in the range of 10–3000 s^–1^ was applied for the viscosity measurement. The measured viscosities are shown in Figure S1A (Supporting Information).

The aqueous debinding step was optimized by measuring the speed of PEG removal in dependence of debinding duration and temperature of the debinding water bath. Green parts were weighed and then immersed in water at 20 and 40 °C for various debinding durations. After drying the debinded samples, the samples were weighed again to determine the change in mass. The mass loss was then compared to the theoretical PEG amount in the feedstock (see Figure S1B, Supporting Information).

Thermogravimetric analysis (TGA) was used to analyze the thermal degradation of the composite allowing optimization of the thermal debinding process parameters. For this the aqueous debinded MAS composite were characterized using a TGA of type STA449F5 (Netzsch, Germany). The samples were heated up to 600 °C in air using heating rates of 3 K min^‐1^ while the mass loss was recorded. The TGA measurement is shown in Figure S1C (Supporting Information).

### Characterization of MAS Ceramics

Density *ρ* was measured using the Archimedes principle using a lab scale lab scale Quintix 124‐1S and a density kit analytical balance YDK03 (Satorius AG, Germany). For this the pre‐sintered or HIP treated MAS was first weighed in the dry state (*m*) and then immersed in DI water (*T* = 22°C) with a small amount of soap to determine the buoyancy mass *m*_b_. The density was then calculated using the following equation with *ρ*_H2O_ being the density of water. The measured data was shown in Table S1 (Supporting Information).

(1) $$\rho =\rho_{H2O}m/m_{b}$$

The shrinkage was analyzed by measuring the dimensions of injection molded green parts and HIP treated transparent MAS ceramics using a caliper. The measurement data is shown in Table S2 (Supporting Information). Theoretical shrinkage *Y_s_* can be calculated in dependence of the solid loading *Φ*, theoretical density *ρ_t,_* and final density *ρ_f_* of the manufactured object using following equation.

(2) $$Y_{s}=1-{\left(\phi /\left(\rho_{t}/\rho_{f}\right)\right)}^{1/3}$$

Chemical resistance of transparent MAS ceramics was characterized by immersing the transparent HIP treated ceramics into acidic, basic, and organic chemicals for 24 h. Afterward the parts were cleaned with water and carefully dried using paper tissue. The parts were weighed before and after the immersion in order to calculate the mass loss. The results are shown in Table S4 (Supporting Information).

Total transmission was measured using a UV–vis‐NIR spectrometer of type UV–3600i Plus (Shimadzu, Japan) over a range from 200 to 2500 nm equipped with an integrating sphere attachment of type ISR‐1503 (Shimadzu, Japan). An injection molded and HIP treated MAS plate with a thickness of 1.8 mm was used for the measurement. The theoretical maximum transmission *T*_max_ was calculated from the refractive index *n* using following equation. ^[^ ^16^, ^43^ ^]^

(3) $$T=2n/(n^{2}-1)$$

The refractive index, as a function of the wavelength *λ*, was calculated using the *Sellmeier* equation. ^[^ ^16^, ^43^ ^]^

(4) $$n^{2}-\lambda \;=\;1.8938\;\lambda^{2}\;/\;\left(\lambda^{2}-0.09942^{2}\right)\;+\;3.0755\;\lambda^{2}\;/\;\left(\lambda^{2}-15.826^{2}\right)\;\;\;$$

A Fourier transform infrared (FTIR) spectrometer of type Frontier 100 MIR‐FTIR (Perkin Elmer, Germany) was used to measure inline transmission of a 1.8 mm thick injection molded and HIP treated MAS plate in the mid‐infrared region from 3500 up to 7000 nm.

An Atomic force microscope (AFM) of type Multimode 8 (Bruker, Germany) was used to analyze the surface quality of the mold, pre‐sintered and HIP treated MAS on a surface area of 100 µm^2^. All samples were measured as prepared without further post‐processing steps.

White light microscopy of HIP treated MAS was performed using a white light interferometer NewView 9000 (Zygo, USA). The software Gwyddion V2.61 was used to extract height profiles and calculate the surface roughness.

The refractive indices at a wavelength of 435.8 (*n*_g_), 486.1 (*n*_F_), and 546.1 nm (*n*_e_) were measured using a refractometer of type ATR‐L (Schmidt+Haensch, Germany) at a temperature of 20 °C. For the measurement the transparent HIP treated MAS were immersed in diiodomethane (Sigma–Aldrich, Germany). A minimum of nine measurements were conducted for each wavelength. The average and standard deviation of the measured refractive indices were shown in Table S8 (Supporting Information). The refractive index *n*(*λ*) in the visual wavelength region (400–700 nm) was approximated by fitting the measurements using the *Cauchy* equation: ^[^ ^24^, ^42^ ^]^

(5) $$n\left(\lambda \right)\;=\;A\;+\;B/\lambda^{2}\;+\;C/\lambda^{4}\;$$

As a simplification the parameter C, responsible for controlling the curvature at low wavelengths, was approximated to be close to zero and therefore neglected for fitting the refractive index in the visible region (400–700 nm). ^[^ ^42^ ^]^ As fitting parameters, we obtained A = 1.6957 ± 0.0005 and B = 7290 ± 140 nm^2^ for HIP treated MAS. The Abbe number *ν* was derived from the fitted data using the refractive indices at wavelengths of 486.1 (*n*_F_), 587.6 (*n*_d_), and 656.3 nm (*n*_C_) using the following equation:

(6) $$n\;=\;\left(n_{d}\;-\;1\right)\;/\left(n_{F}\;-\;n_{C}\right)$$

Micro‐optical structures in transparent HIP treated MAS as well as grain sizes and grain size distribution of pre‐sintered MAS were analyzed using scanning electron microscopy (SEM) of type Quanta FEG 250 (FEI Instruments, USA). For grain size analysis, the samples were ground, polished, and thermally etched for 30 min at 1400 °C beforehand. Grain size analysis of HIP treated MAS samples was done using an optical microscope of type VHX 6000 (Keyence Corporation, Japan) without any post processing. To determine mean grain diameters a minimum of 100 grains were manually measured for each sample using the software *ImageJ*.

Vickers Hardness (*HV0.1*) of transparent MAS ceramics was measured using a Micro‐Vickers Hardness Tester of type Falcon 608 (Innovatest, Netherlands). For the measurement a load of 100 mN was applied over a duration of 10 s. An injection molded and HIP treated MAS sample having a thickness of 1 mm was used for the measurement and a commercial transparent MAS single crystal substrate with 1 mm thickness (Crystal GmbH, Germany) was used as reference.

Bending strength was measured by three‐point bending on a Zwick model Z005 (Zwick Roell, Germany). Transparent MAS bars with a thickness of 1.43 mm and a width of 4.25 mm prepared by injection molding and subsequent sintering and HIP treatment were used for the measurement. The bars were placed on supports with a distance of 25.2 mm and a load was applied in the middle using a load speed of 0.5 mm min^–1^ at room temperature until sample breakage. Data recording and analyzation were done using the software Zwick TestXpert II.

The X‐Ray Fluorescence (XRF) of transparent MAS components was measured using a spectrometer of type µ‐XRF M4 Tornado (Bruker Corporation, MA, USA). The measurement was performed in vacuum using a 50 kV beam at 200 µA. The sample was measured for a duration of 30 s.

X‐Ray Diffraction (XRD) of HIP treated MAS was recorded on a diffractometer of type D8 Discover (Bruker AXS GmbH, Germany) in Bragg‐Brentano geometry using a LynxEye XE‐T detector and a Cu‐K*α* radiation source (0.154060 nm). A MAS reference diffractogram taken from a public database (RRUFF ID: R050392.1) was used as a comparison.

SEM‐EDX measurements of transparent MAS samples were conducted on a SEM of type Scios 2 DualBeam (Thermo Fisher Scientific, Germany) equipped with an Octane Elite EDS system (EDAX, Germany).

The focal lengths *f* of the spherical MAS microlenses and the macroscopic lens were calculated according to the lensmaker equation (7), with *R* the radius of the lens curvature and *n* the refractive index of MAS.^[^ ^44^ ^]^

(7) $$f\;=\;R\;/\;\left(n-1\right)$$

The curvature *R* of the spherical lens was calculated from the center height of the lens *h* and the lens radius *r* according to equation (8).^[^ ^44^ ^]^ The lens dimensions *h* and *r* were determined from surface profiles of white light interferometry measurements for the microlenses or by caliper measurements for the macroscopic lens.

(8) $$R\;=\;h\;/2\;+\;r^{2}\;/2h$$

The roughness of spherical MAS microlenses was determined from white light interferometry measurements. For this, the measured height profile was fitted to a 2nd order polynomial equation showing a good approximation of the lens curvature. The fitted data was subtracted from the measured profile. The roughness *R*_q_ was then defined as the standard deviation of the subtracted curve. The measured and processed data was shown in Figure S6 (Supporting Information).

## Conflict of Interest

The authors declare no conflict of interest.

## Supporting information

Supporting Information

## Data Availability

The data that support the findings of this study are available in the supplementary material of this article.

## References

[1] M. Rubat du Merac , H.‐J. Kleebe , M. M. Müller , I. E. Reimanis , J. Am. Ceram. Soc. 2013, 96, 3341.

[2] Z. Xiao , S. Yu , Y. Li , S. Ruan , L. B. Kong , Q. Huang , Z. Huang , K. Zhou , H. Su , Z. Yao , W. Que , Y. Liu , T. Zhang , J. Wang , P. Liu , D. Shen , M. Allix , J. Zhang , D. Tang , Mater. Sci. Eng. R: Rep. 2020, 139, 100518.

[3] A. Goldstein , A. Krell , J. Am. Ceram. Soc. 2016, 99, 25.

[4] A. Krell , J. Klimke , T. Hutzler , J. Eur. Ceram. Soc. 2009, 29, 275.

[5] G. Villalobos , S. Bayya , W. Kim , C. Baker , J. Sanghera , M. Hunt , B. Sadowski , F. Miklos , I. Aggarwal , J. Mater. Res. 2014, 29, 2266.

[6] A. A. DiGiovanni , L. Fehrenbacher , D. W. Roy , in (Ed.: R. W. Tustison ), Orlando, Florida, USA, 2005, 56.

[7] A. Jouini , A. Yoshikawa , A. Brenier , T. Fukuda , G. Boulon , phys. stat. sol. (c) 2007, 4, 1380.

[8] M. Krishnan , B. Tiwari , S. Seema , N. Kalra , P. Biswas , K. Rajeswari , M. B. Suresh , R. Johnson , N. M. Gokhale , S. R. Iyer , S. Londhe , V. Arora , R. P. Tripathi , J. Mater. Sci.: Mater. Med. 2014, 25, 2591. 25027301 10.1007/s10856-014-5268-3

[9] A. Ibarra , E. R. Hodgson , Nucl. Instrum. Methods Phys. Res. B 2004, 218, 29.

[10] A. Krell , T. Hutzler , J. Klimke , J. Eur. Ceram. Soc. 2009, 29, 207.

[11] Y. Ramisetty , S. Sastri , U. Kashalikar , M. Goldman , N. Nag , Am. Ceram. Soc. Bull 2013, 92, 20.

[12] P. Hartmann , R. Jedamzik , S. Reichel , B. Schreder , Appl. Opt. 2010, 49, D157.

[13] Z. Shi , Q. Zhao , B. Guo , T. Ji , H. Wang , Mater. Des. 2020, 193, 108858.

[14] I. Ganesh , Int. Mater. Rev. 2013, 58, 63.

[15] I. Reimanis , H.‐J. Kleebe , J. Am. Ceram. Soc. 2009, 92, 1472.

[16] H. Wang , L. Y. Liu , P. Ye , Z. Huang , A. Y. R. Ng , Z. Du , Z. Dong , D. Tang , C. L. Gan , Adv. Mater. 2021, 33, 2007072. 10.1002/adma.202007072 33682251

[17] J. M. Pappas , X. Dong , Materials 2020, 13, 4810. 10.3390/ma13214810 PMC7663333 33126542

[18] J. W. Oh , C. W. Gal , D. Shin , J. M. Park , W. S. Yang , S. J. Park , J. Jpn. Soc. Powder Powder Metall. 2018, 9, 539.

[19] L. Nyborg , E. Carlström , A. Warren , H. Bertilsson , Powder Metall. 1998, 41, 41.

[20] Z. S. Rak , Powder Metall. Met. Ceram. 1999, 38, 126.

[21] M. Mader , O. Schlatter , B. Heck , A. Warmbold , A. Dorn , H. Zappe , P. Risch , D. Helmer , F. Kotz , B. E. Rapp , Science 2021, 372, 182. 33833122 10.1126/science.abf1537

[22] I. Todd , A. T. Sidambe , in Adv. Powder Metall., Elsevier, Amsterdam, 2013, 109.

[23] G. Wen , P. Cao , B. Gabbitas , D. Zhang , N. Edmonds , Metall and Mat Trans A 2013, 44, 1530.

[24] I. Yamashita , M. Kudo , K. Tsukuma , TOSOH Res. Technol. Rev 2012, 56, 11.

[25] W. Liu , Z. Xie , G. Liu , X. Yang , J. Am. Ceram. Soc. 2011, 94, 3211.

[26] J. W. Oh , W. S. Lee , S. J. Park , Powder Technol. 2017, 311, 18.

[27] J. M. Park , J. S. Han , J. W. Oh , S. J. Park , Met. Mater. Int. 2019, 27, 1069.

[28] K. Tsukuma , J. Ceram. Soc. Jpn. 2006, 114, 802.

[29] H.‐Y. Lee , J.‐S. Kim , N.‐M. Hwang , D.‐Y. Kim , J. Eur. Ceram. Soc. 2000, 20, 731.

[30] A. Krell , J. Klimke , T. Hutzler , Optical Materials 2009, 31, 1144.

[31] A. Witek , Materia ly Ceramiczne 2013, 65, 386.

[32] A. Goldstein , J. Eur. Ceram. Soc. 2012, 32, 2869.

[33] M. Sokol , B. Ratzker , S. Kalabukhov , M. P. Dariel , E. Galun , N. Frage , Adv. Mater. 2018, 30, 1706283. 10.1002/adma.201706283 29920779

[34] A. Krell , A. Bales , Int. J. Appl. Ceram. Technol. 2011, 8, 1108.

[35] A. Rothman , S. Kalabukhov , N. Sverdlov , M. P. Dariel , N. Frage , Int. J. Appl. Ceram. Technol. 2014, 11, 146.

[36] I. Ganesh , G. Jaganatha Reddy , G. Sundararajan , S. M. Olhero , P. M. C. Torres , J. M. F. Ferreira , Ceram. Int. 2010, 36, 473.

[37] C. Gajdowski , J. Böhmler , Y. Lorgouilloux , S. Lemonnier , S. d'Astorg , E. Barraud , A. Leriche , J. Eur. Ceram. Soc. 2017, 37, 5347.

[38] N. Frage , S. Cohen , S. Meir , S. Kalabukhov , M. P. Dariel , J. Mater. Sci. 2007, 42, 3273.

[39] V. V. Osipov , V. A. Shitov , R. N. Maksimov , K. E. Lukyashin , V. I. Solomonov , A. V. Ishchenko , J. Am. Ceram. Soc. 2019, 102, 4757.

[40] L. Basyrova , S. Balabanov , A. Belyaev , V. Drobotenko , A. Volokitina , V. Vitkin , O. Dymshits , P. Loiko , Journal of Physics 2019, 1410, 5.

[41] T. Mroz , L. M. Goldman , A. D. Gledhill , D. Li , N. P. Padture , Int. J. Appl. Ceram. Technol. 2012, 9, 83.

[42] J. Orava , J. Šik , T. Wágner , M. Frumar , J. Appl. Phys. 2008, 103, 083512.

[43] E. D. Palik , Handbook of Optical Constants of Solids, Elsevier, Amsterdam, The Netherlands, 1991.

[44] P. Nussbaum , R. Völkel , H. P. Herzig , M. Eisner , S. Haselbeck , Pure Appl. Opt. 1997, 6, 617.

