# Supplementary material for: Injection Molding of Magnesium Aluminate Spinel Nanocomposites for High‐Throughput Manufacturing of Transparent Ceramics
Source: Adv Sci (Weinh). 2022 Sep 4;9(31):2204385. doi: 10.1002/advs.202204385 (PMC9631057; doi:10.1002/advs.202204385)
Supplement: Supplementary file 1 — Supporting Information [file ADVS-9-2204385-s001.pdf]

ADVANCED SCIENCE
Open Access

Supporting Information

for *Adv. Sci.*, DOI 10.1002/advs.202204385

Injection Molding of Magnesium Aluminate Spinel Nanocomposites for High-Throughput Manufacturing of Transparent Ceramics

*Markus Mader, Richard Prediger, Karl G. Schell, Gabriela Schmidt, Alex Dorn, Sophie Jenne, Sebastian Kluck, Leonhard Hambitzer, Manuel Luitz, Claudia Schwarz, Marcel Milich, Christian Greiner, Bastian E. Rapp and Frederik Kotz-Helmer**

Supporting Information

Injection molding of magnesium aluminate spinel nanocomposites for high-throughput manufacturing of transparent ceramics

*Markus Mader, Richard Prediger, Karl. G. Schell, Gabriela Schmidt, Alex Dorn, Sophie Jenne, Sebastian Kluck, Leonhard Hambitzer, Claudia Schwarz, Marcel Milich, Christian Greiner, Bastian E. Rapp, Frederik Kotz-Helmer**

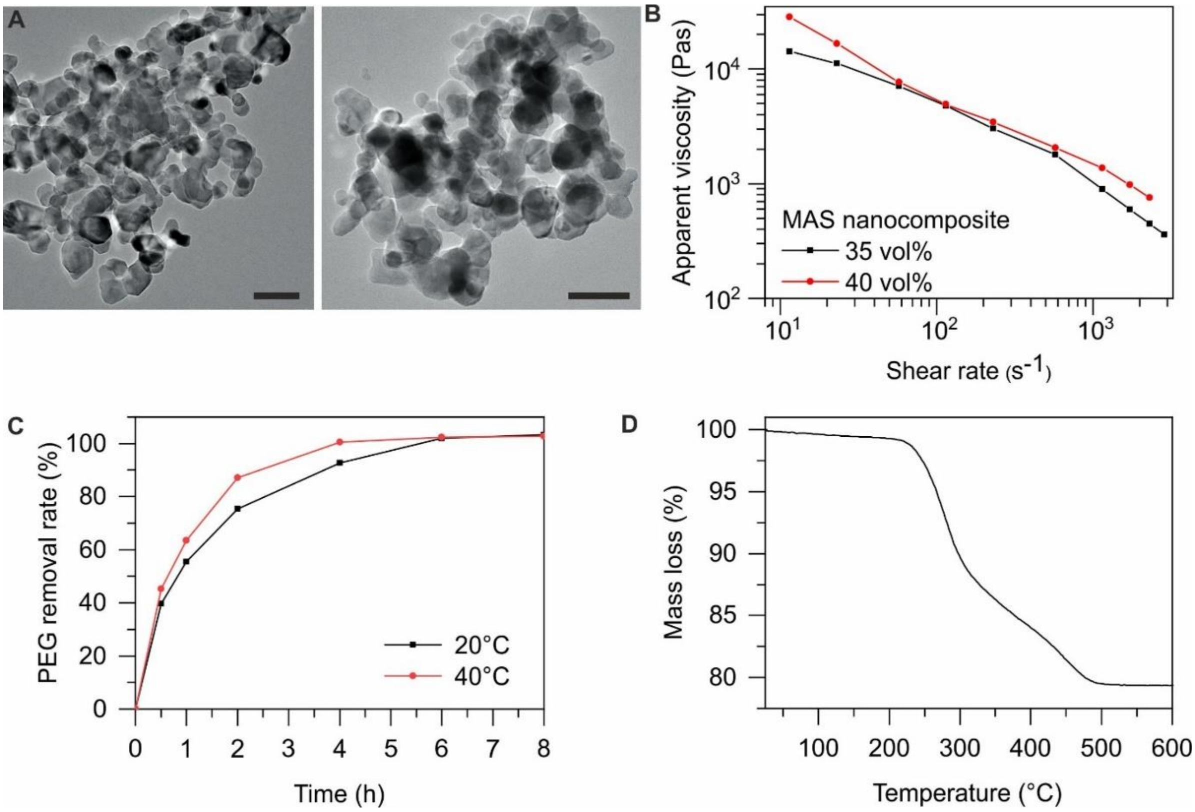

Figure S1: Characterization of MAS nanoparticles and thermoplastic MAS nanocomposite and debinding optimization. (A) Transmission electron microscopy (TEM) images of MAS nanoparticles showing a roughly spherical particle shape and ~50 nm particle diameters. Scale bars: 100 nm. (B) Viscosity measurement of thermoplastic MAS nanocomposites containing 35 vol.% and 40 vol.% MAS nanopowder using capillary rheometer at a temperature of 140 °C. (C) Study of the PEG debinding speed of a 2 mm thick green part during the first aqueous debinding step. The PEG removal rate in water at room temperature (20 °C) and at slightly elevated temperature (40 °C) were determined at different time durations. (D) Thermogravimetric analysis (TGA) measurement of aqueous debinded MAS nanocomposites for optimization of the temperature program used in the second debinding step. Dwelling phases were set at critical decomposition temperatures (270 °C, 400 °C and 600 °C).

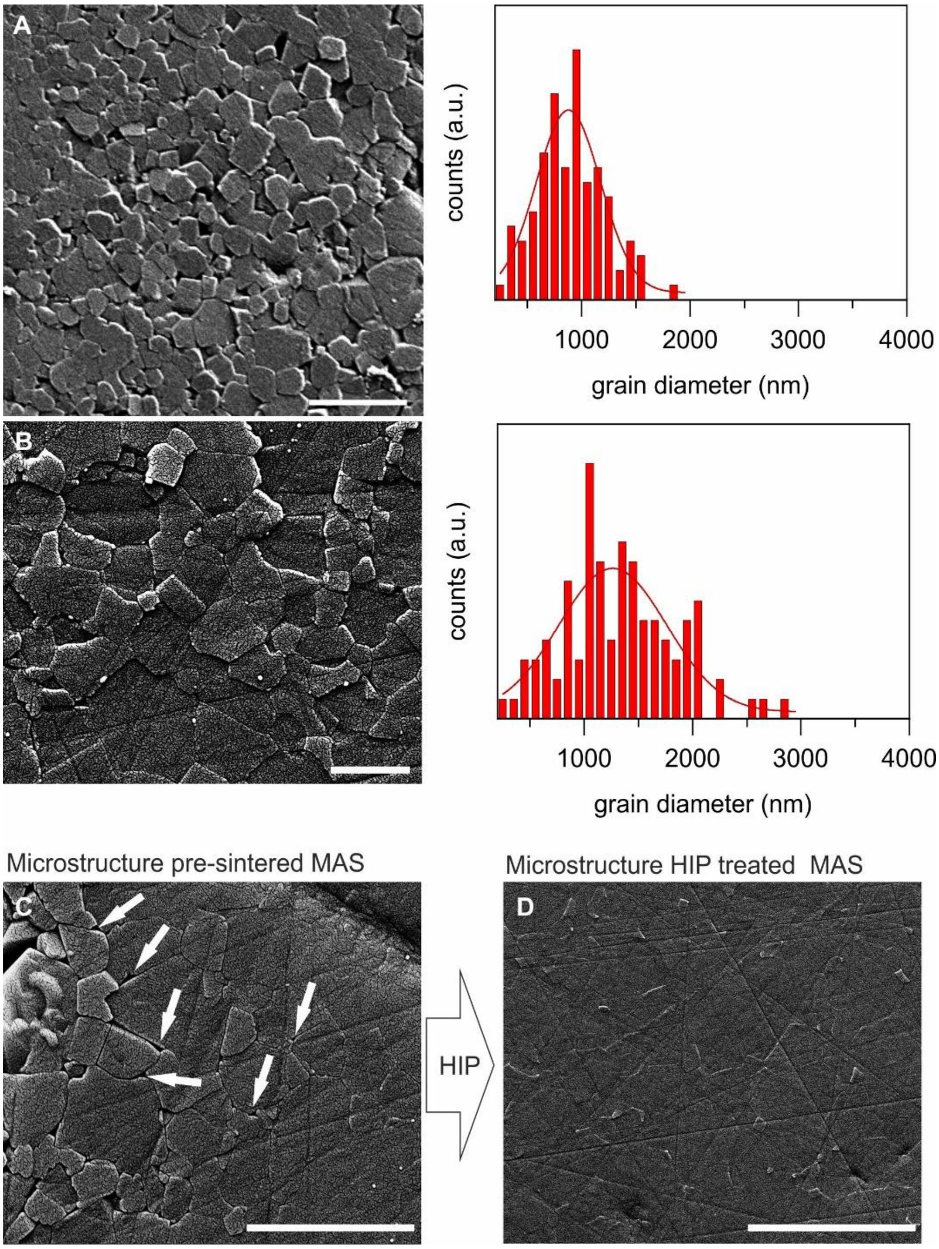

Figure S2: Grain size analysis of pre-sintered MAS ceramics. (A) Exemplary SEM image for grain size analysis of a pre-sintered MAS ceramic fabricated by injection molding of a 35 vol.% nanocomposite and the respective grain size histogram. Scale bar: 2 µm. (B) Exemplary SEM image for grain size analysis of a pre-sintered MAS ceramic fabricated by injection molding of a 40 vol.% nanocomposite and the respective grain size histogram. Scale bar: 2 µm. (C/D) Comparison of the MAS grain microstructure after pre-sintering and after additional HIP treatment. Pre-sintered MAS (C, scale bar: 5 µm) shows residual porosity as indicated by white arrows. After the additional HIP treatment (D, scale bar: 10 µm) the MAS ceramic is fully densified and no porosity can be observed.

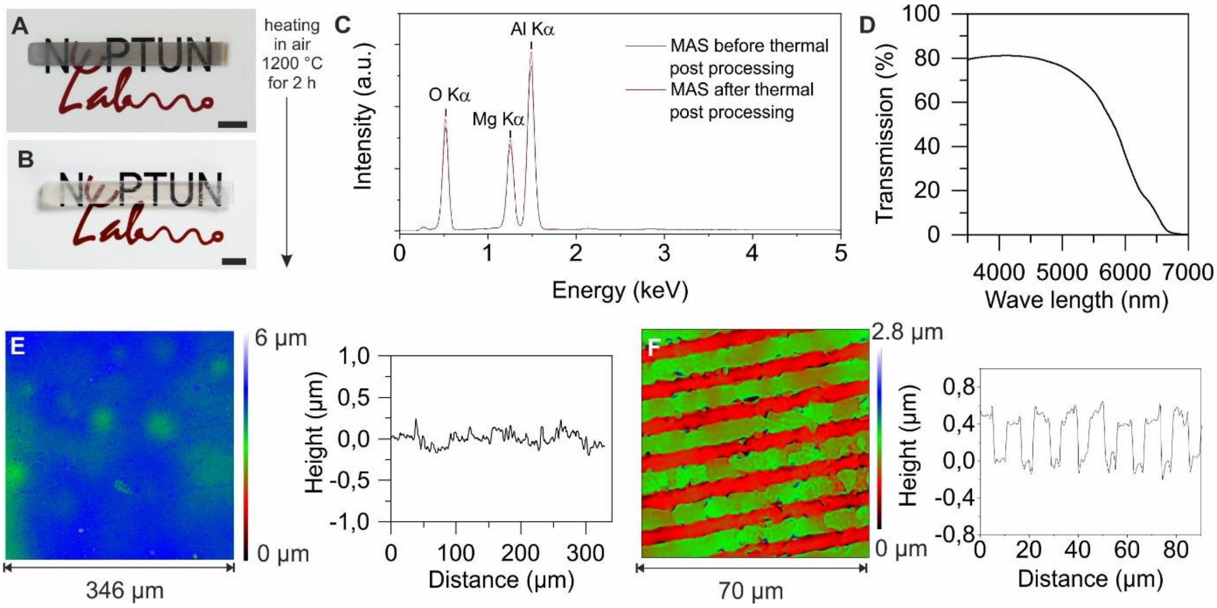

Figure S3: Additional characterization of transparent MAS after HIP treatment. (A) After HIP treatment the MAs ceramics show a process inherent discoloration. Scale bar: 4 mm. (B) SEM-EDX measurement of transparent MAS components after HIP treatment with and without the additional thermal heat treatment to remove discoloration. (C) By heating the HIP treated MAS parts in air to a temperature of 1200 °C for 2 h the discoloration can be removed. Scale bar: 4 mm. (D) FTIR spectra of a 1.8 mm thick transparent MAS plate showing high transmission of ~ 80 % up to 5000 nm. (E) White light microscopy photograph and respective height profile of injection molded and HIP treated MAS showing a surface roughness of S_q = 148 nm on an area of 346 µm x 346 µm. (F) White light microscopy photograph and respective height profile of injection molded and HIP treated microstructured MAS showing a line grid microstructure with line widths of ~6 µm and line heights of ~ 0.4 µm.

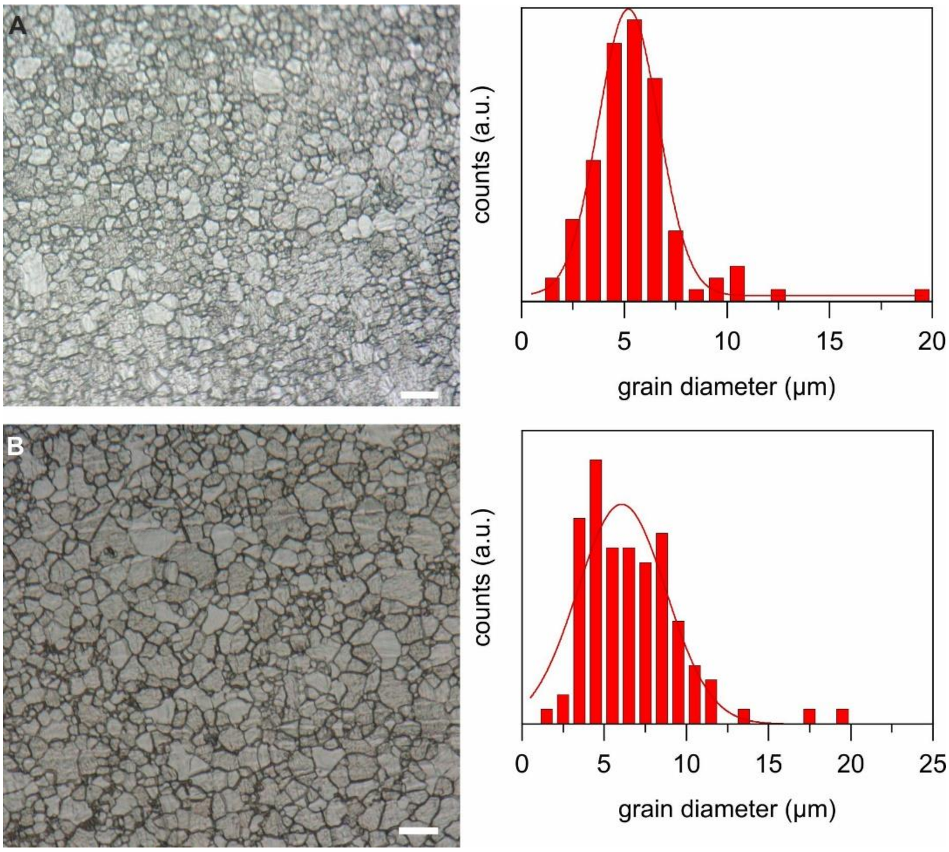

Figure S4: Grain size analysis of HIP treated MAS ceramics. (A) Exemplary microscope image for grain size analysis of a HIP treated MAS ceramic fabricated by injection molding of a 35 vol.% nanocomposite and the respective grain size histogram. Scale bar: 20 µm. (B) Exemplary microscope image for grain size analysis of a HIP treated MAS ceramic fabricated by injection molding of a 40 vol.% nanocomposite and the respective grain size histogram. Scale bar: 20 µm.

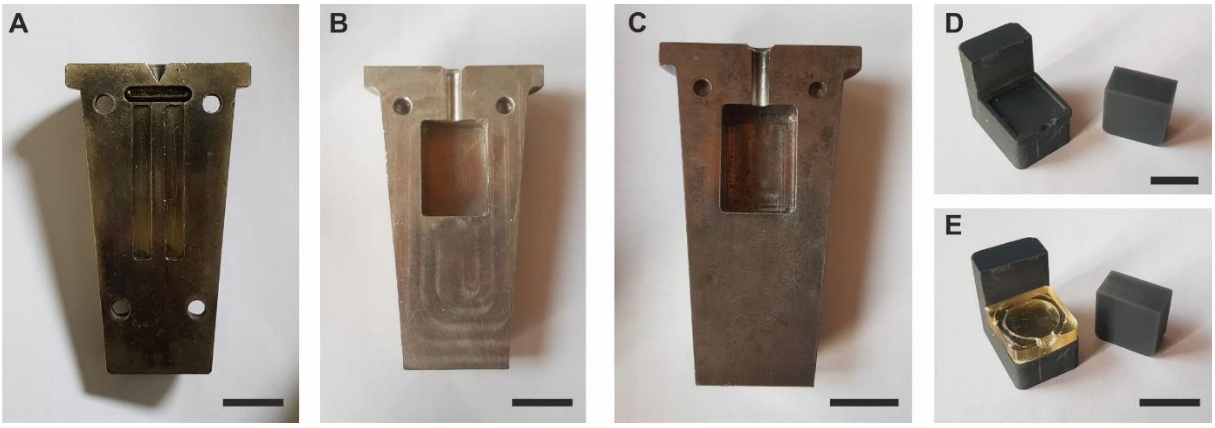

Figure S5: Molding tools used to injection mold the various MAS components shown. (A) Brass mold for bar shaped components. (B) Basic molding tool made from alumina. (C) Basic molding tool made from steel. (D) Mold inserts for the basic molds shown in (B and C) manufactured by stereolithography 3D printing of a commercial tooling resin (ASIGA FusionGray) for injection molding of plates. (E) An epoxy resin mold insert manufactured by replication of a master lens in combination with 3D printed spacers for injection molding of lenses. Scale bars: 20 mm.

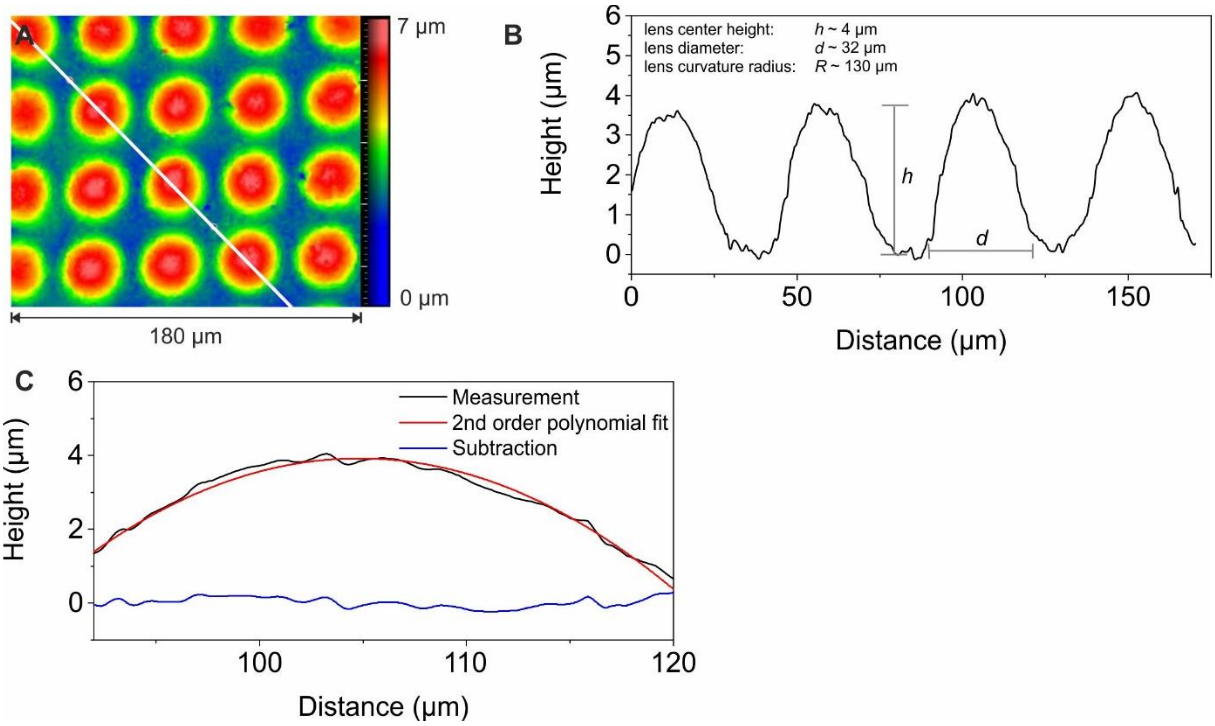

Figure S6: Characterization of MAS microlenses. (A) White light interferometry measurement of the MAS spherical microlenses and the corresponding height profile (B) for determination of lens height, diameter and curvature radius (according to equation (8)). (C) Calculation of the surface roughness of the MAS microlenses. For this the measurement (black curve) was fitted by a 2nd order polynomial fit (red curve). The surface roughness R_q was extracted as standard deviation of the subtraction (blue curve) of fitted data from the measurement (R_q ~ 150 nm).

Table S1: Density measurement of HIP treated transparent MAS components. Measured data for the density calculation using the Archimedes principle. Three different pre-sintered and HIP treated MAS components were measured to show that full density is achieved. Each sample was measured three times.

No.	Dry mass [g]			Buoyancy mass [g]			Calculated density [g cm^{-3}]		
	m1	***m2***	***m3***	***m_b1***	***m_b1***	***m_b1***	***r1***	***r2***	***r3***
1	0.3362	0.3362	0.3362	-0.0934	-0.0940	-0.0939	3.5917	3.5687	3.5725
2	0.7816	0.7816	0.7815	-0.2173	-0.2174	-0.2175	3.5890	3.5873	3.5852
3	0.8997	0.8897	0.8898	-0.2506	-0.2506	-2.0509	3.5823	3.5823	3.5784

Table S2: Quantitative analysis of SEM-EDX measurements of transparent MAS. Normalized quantities of the elements found in the SEM-EDX measurement.

**Element**	**Norm. at.%**<br>Before thermal post process	**Norm. at.%**<br>After thermal post process
Mg	14.4 ± 5	14.1 ± 5
Al	29.4 ± 5	29.0 ± 5
O	52.3 ± 7	52.1 ± 7
C	3.9 ± 18	4.8 ± 16

Table S3: Shrinkage measurement of injection molded MAS during the sintering steps. Measured data for shrinkage calculation from green part (35 vol.% feedstock) to HIP treated MAS ceramic with full density. Three different injection molded green parts and the respective HIP treated MAS were measured along x-y as well as z-axis to show isotropic shrinkage.

No.	Green part [mm]		HIP treated part [mm]		Linear shrinkage [%]	
	x-y	*z*	*x-y*	*z*	Y_{x-y}	Y_z
1	6.11	2.16	4.30	1.53	29.62	29.17
2	6.11	2.15	4.31	1.51	29.46	29.76
3	6.10	2.16	4.30	1.52	29.51	29.63

Table S4: Chemical resistance of injection molded transparent MAS. Mass changes of transparent MAS components immersed for 24 h in various chemicals showing that the ceramics have high chemical resistance. The measurement error on the weights is ± 0.1 mg, respectively.

Chemical	Mass before immersion /mg	Mass after immersion /mg	Mass change /%	Visual change
HCl 1M	788.3	788.4	0.01 ± 0.02	None
NaOH 1M	886.5	886.4	-0.01 ± 0.02	None
NH_3 (25%)	798.6	798.7	0.01 ± 0.02	None
$CHCl_3$	793.2	793.1	-0.01 ± 0.02	None
Toluene	949.5	949.6	-0.01 ± 0.02	None
Acetone	780.3	780.3	0 ± 0.02	None
Dimethylsulfoxid	890.2	890.2	0 ± 0.02	None

Table S5: Quantitative analysis of XRF measurement of transparent MAS. Normalized quantities of the elements found in the XRF measurement by weight (wt%) and number (mol.%).

Element	Norm. wt.%	Norm. mol.%	Deviance mol.%
Mg	25.642	27.72	0.13
Al	74.253	72.23	0.17
Cr	0.013	0.0067	0.0003
Fe	0.092	0.0432	0.0006

Table S6: Measurement of Vickers hardness *HV0.1* of transparent MAS components. Measured Vickers hardness values using a load of 100 mN and a load duration of 10 s. An injection molded and HIP treated MAS samples (1 mm thickness) was measured in comparison to a MAS substrate (1 mm thickness, single crystal).

**No.**	***HV0.1* (commercial MAS)**	***HV0.1* (Injection molded and HIP treated MAS)**
1	1508.81	1924.30
2	1481.93	1897.86
3	1455.12	1881.60
4	1507.57	1810.54
5	1508.00	1856.42
6	1495.19	1757.77
7	1508.31	1665.94
8	1536.87	1671.15
9	1475.28	1940.63
10	1522.87	1911.08
11	1487.23	2060.99
12	1468.67	1661.59
13	1508.81	1925.48
14	1495.25	1971.95
15	1508.87	1955.11
Average	1500	1860
Standard deviation	20	120

Table S7: Bending strength f_m of transparent MAS components. Measured bending strength f_m values of transparent MAS components by 3-point bending at room temperature.

**No.**	**f_m [MPa]**
1	217.413
2	121.421
3	139.629
4	234.200
5	145.045
6	161.716
7	155.865
8	134.865
Average	164
Standard deviation	40

Table S8: Refractive index measurement of transparent MAS. The refractive index of the injection molded and HIP treated MAS was measured at 486.1 nm, 546.1 nm and 587.6 nm to determine $n_{g,20}$, $n_{F,20}$ and $n_{e,20}$ (average with standard deviation, min. 9 measurements each). The measurements were fitted using the *Cauchy* equation (equation (5)) to calculate $n_{d,20}$ (587.5 nm), $n_{D,20}$ (589.3 nm) and $n_{C,20}$ (656.3 nm) in order to analyze the optical dispersion. The error on the fitted refractive indices was calculated by error propagation of the *Cauchy* equation. The theoretically calculated value according to equation (4) are shown as comparison.

Wave length [nm]	Measured *n* (HIP MAS)	Fitted *n* (HIP MAS)	Theoretical *n*
435.8 (n_g)	1.7341 ± 0.0010	1.7341 ± 0.0009	1.7307
486.1 (n_F)	1.7260 ± 0.0019	1.7265 ± 0.0008	1.7244
546.1 (n_e)	1.7201 ± 0.0002	1.7201 ± 0.0007	1.7190
587.6 (n_d)	-	1.7168 ± 0.0006	1.7162
589.3 (n_D)	-	1.7167 ± 0.0006	1.7161
656.3 (n_C)	-	1.7126 ± 0.0006	1.7126
